# *Salicornia strobilacea* (Synonym of *Halocnemum strobilaceum*) Grown under Different Tidal Regimes Selects Rhizosphere Bacteria Capable of Promoting Plant Growth

**DOI:** 10.3389/fmicb.2016.01286

**Published:** 2016-08-22

**Authors:** Ramona Marasco, Francesca Mapelli, Eleonora Rolli, Maria J. Mosqueira, Marco Fusi, Paola Bariselli, Muppala Reddy, Ameur Cherif, George Tsiamis, Sara Borin, Daniele Daffonchio

**Affiliations:** ^1^Biological and Environmental Sciences and Engineering Division, King Abdullah University of Science and Technology, ThuwalSaudi Arabia; ^2^Department of Food, Environmental and Nutritional Sciences, University of Milan, MilanItaly; ^3^Greenhouse Laboratory, King Abdullah University of Science and Technology, ThuwalSaudi Arabia; ^4^Institut Supérieur de Biotechnologie Sidi Thabet, BVBGR-LR11ES31, Manouba University, ArianaTunisia; ^5^Department of Environmental and Natural Resources Management, University of Patras, Panepistimioupoli PatronGreece

**Keywords:** plant growth promoting bacteria, bacterial diversity, salicornia, tidal regime, coastal environments, halophyte, salt systems

## Abstract

Halophytes classified under the common name of salicornia colonize salty and coastal environments across tidal inundation gradients. To unravel the role of tide-related regimes on the structure and functionality of root associated bacteria, the rhizospheric soil of *Salicornia strobilacea* (synonym of *Halocnemum strobilaceum*) plants was studied in a tidal zone of the coastline of Southern Tunisia. Although total counts of cultivable bacteria did not change in the rhizosphere of plants grown along a tidal gradient, significant differences were observed in the diversity of both the cultivable and uncultivable bacterial communities. This observation indicates that the tidal regime is contributing to the bacterial species selection in the rhizosphere. Despite the observed diversity in the bacterial community structure, the plant growth promoting (PGP) potential of cultivable rhizospheric bacteria, assessed through *in vitro* and *in vivo* tests, was equally distributed along the tidal gradient. Root colonization tests with selected strains proved that halophyte rhizospheric bacteria (i) stably colonize *S. strobilacea* rhizoplane and the plant shoot suggesting that they move from the root to the shoot and (ii) are capable of improving plant growth. The versatility in the root colonization, the overall PGP traits and the *in vivo* plant growth promotion under saline condition suggest that such beneficial activities likely take place naturally under a range of tidal regimes.

## Introduction

Halophilic plants classified under the common name of salicornia (*Chenopodiaceae*) have been proposed as model plants to investigate salt adaptation (Feng et al., 2015). *Salicornia* species have great potential for phytoremediation of saline soils (Al-Mailem et al., 2010; Oliveira et al., 2014) and mariculture eﬄuent (Shpigel et al., 2013) and for oilseed production (Glenn et al., 1991; Attia et al., 1997), and can be cultivated in areas of negligible interest for agriculture.

*Salicornia* plants are common in many saline ecosystems, comprising dry salt lakes, lagoons, and coastal areas where they are exposed to variable tidal regimes (i.e., subtidal, intertidal, and supratidal). Soil and sediment bacterial communities are subjected to the influence of multiple environmental parameters that shape their composition in terms of taxa and their relative abundance (Fierer and Jackson, 2006; Fierer et al., 2012; Dini-Andreote et al., 2014), especially under “extreme” physico-chemical conditions (CAREX, 2011; Marteinsson et al., 2013; Borruso et al., 2014). Instead, rhizosphere is a particular habitat where plant root exudates can gradually alters the sediment conditions to select and enrich a specific rhizo-microbiome (Berg and Smalla, 2009; Dennis et al., 2010; Lundberg et al., 2012; Marasco et al., 2013a). In coastal environments the rhizosphere soil/sediments are subjected to the constant selective pressure imposed by the plant roots, but to different stressor intensity depending on the type of tidal regime. The capacity of salicornia to flourish under variable tidal conditions, including waterlogged soil, provides the unique opportunity to study tide influence on the microbiome structure.

Tidal events determine the concomitant shifts of oxygen and nutrient availability, temperature, and salinity in the soil/sediments (Alongi, 1988; Mitra et al., 2008; Mendes et al., 2014; Wang et al., 2015), implying that the ability to cope with cyclic variation of such multiple environmental factors is crucial for the establishment of microbial communities.

Besides contributing to the balance of carbon, nitrogen, and other elements in coastal ecosystems (Kirchman et al., 1984; Chauhan et al., 2009; Zhang et al., 2014), bacteria associated to plant roots growing in such environments are adapted to survive under saline condition and determine direct and indirect plant growth promoting (PGP) effects that strongly affect the fertility of the soil/sediment substrate and stimulate plant growth (Hildebrandt et al., 2001; Rodriguez et al., 2008; Siddikee, 2010; Gontia et al., 2011; Jha et al., 2012; Mapelli et al., 2013a; El-Sayed et al., 2014; Mesa et al., 2015). However, little information is available on the role of tidal flooding regimes on determining the structure and diversity of plant-associated bacterial communities and their functional PGP potential.

Here, we studied the bacterial microbiome associated to *Salicornia strobilacea* (synonym of *Halocnemum strobilaceum*) plants grown under different tidal regimes in a coastal area located in Southern Tunisia aiming to assess (i) the role of tide on the selection of rhizospheric bacterial assemblages, (ii) the ability of selected rhizosphere bacterial types to actively recolonize *S. strobilacea* roots, and (iii) the capacity of such bacteria to promote plant growth under saline conditions. Since the interaction of edaphic and host-plant factors deeply affect bacterial community structure and composition (Panke-buisse et al., 2015; Lareen et al., 2016 and references therein), but rhizosphere microbiome PGP properties are essential for conditioning the soil/sediment for plant establishment, we hypothesize that the tidal regime drives the phylogenetic composition of *S. strobilacea* rhizosphere community, still maintaining the overall PGP potential of such communities (Marasco et al., 2013b).

## Materials and Methods

### Site Description and Soil Sampling

The studied site was located in the coastal area of Ras Lamsa, near Zarzis (N 33°23′21.85′, E 11°7′10.19 55.745′), Tunisia (Supplementary Figures 1A,B). The area was subjected to a constant tidal flooding (Supplementary Figure 2A) with an excursion ranging from 20 cm during neap tide up to 1 m during spring tide. These fluctuations generated three different tidal zones (subtidal, intertidal, and supratidal; Supplementary Figures 1C–F). Climate parameters are reported in the Supplementary Figure 2B. The soil salinity in the selected area was determined in the field with a handheld multi-parameter system and ranged from 33.7 to 36.3 psu (practical salinity units). During the sampling campaign BioDesert III (February 2008), salicornia plants growing in different tidal zones occurring at the studied site were identified. According to morphology, the plants were identified as *S. strobilacea* (synonym of *H. strobilaceum*)^1^ a widespread halophyte in Southern Tunisia (Le Floc’h et al., 2010). The *S. strobilacea* root system (Supplementary Figure 1G) was sampled in triplicate from plants growing on saline soil continuously or partially flooded by tide (“subtidal” and “intertidal,” respectively) or never exposed to tidal flow (“supratidal”). In laboratory the rhizosphere soil – defined as soil particles tightly adhering to roots (1–3 mm) – was separated from the root system as described in Marasco et al. (2012) and stored at -20°C for molecular analyses and at 4°C for microbiological isolation.

### Bacteria Isolation, Genotypic Characterization, and Identification

One gram of rhizospheric soil was suspended in 9 ml of physiological solution (9 g/L NaCl) and shaken for 15 min at 200 rpm at room temperature. The suspension was diluted in 10-fold series and plated onto R2A at three different salinities (5, 10, and 20% NaCl) and King’s B (KB) medium (Oxoid). After 72 h of incubation at 30°C, colony forming units (CFU) per gram were determined. About 25 colonies per each rhizospheric soil sample were randomly selected from the R2A 5% and KB plates, and spread three times on the original medium to obtain pure isolates. A total of 140 pure strains were collected and stored in 25% glycerol at -80°C until use. The DNA was extracted from each isolate by boiling lysis and used to de-replicate the isolates collection using the intergenic transcribed spacers (ITS)-PCR fingerprinting protocol (Ferjani et al., 2015). One representative strain from each ITS-genotype was identified by partial sequencing of the 16S rRNA gene at Macrogen (South Korea) following the protocol described in (Marasco et al., 2013a). To resolve the distribution of the isolate the profile network was used. The network was constructed using Cytoscape version 3.2.1 (Shannon et al., 2003) feed with the table resulted by the command *make_otu_network.py* in Qiime. The biom-table used to build the network was elaborated considering the 16S rRNA sequences of the isolates as OTU assigned with the 97% of similarity using Qiime (Caporaso et al., 2010). The analysis take into account the abundance of each OTU in the samples. Sequences were deposited in the ENA database under accession numbers LN995411– LN995510.

### DNA Extraction and ARISA-PCR

DNA was extracted from 0.5 g of soil using the protocol described in Mapelli et al. (2013a). DNA was quantified using NanoDrop 1000 spectrophotometer (Thermo Scientific, Waltham, MA, USA). The ARISA fingerprinting was obtained using the primer set ITSF, 5′-GTCGTAACAAGGTAGGCCGTA-3′ and ITSReub, 5′-GCCAAGGCATCCACC-3′, as described elsewhere (Cardinale et al., 2004; Fodelianakis et al., 2015). The output peak matrix was transferred to Microsoft Excel for the following analysis. Peaks showing a height value lower than 50 fluorescence units were removed from the output peak matrix before statistical analyses. To account for variability in size associated with standards, ARISA fingerprints were binned ±1 bp from 150 to 300 bp, ±3 bp from 300 to 500 bp and ±10 bp > 500 bp (Mapelli et al., 2013b). Downstream statistical analyses (Principal Coordinates Analysis, PERMANOVA) were performed on the ARISA quantitative dataset, which account for the relative abundance of each peak, by PRIMER v. 6.1, PERMANOVA+ for PRIMER routines (Anderson et al., 2008) and PAST software. The intersections between the three tidal areas have been calculated with the Veen Diagrams software^2^ using as input the ARISA matrixes obtained from the three replicates.

### *In vitro* Screening of PGP Activities and Resistance to Abiotic Stresses

The 100 bacterial strains obtained after de-replication were screened for their ability to growth at different concentration of salt (0, 5, and 10% NaCl). Furthermore, the isolated strains have been screened *in vitro* for several PGP traits, including (i) production of IAA and (ii) siderophores, (iii) mineral phosphate solubilization, (iv) EPS and for tolerance to (v) abiotic stresses (osmotic and temperature variation) following the methods described in (Cherif et al., 2015). The PGP tests were performed both in absence and presence (5%) of sodium chloride.

### Recolonization of *S. strobilacea* Plantlets

A mini-Tn7 transposon system was used for chromosomally fluorescence-tagging halotolerant isolates by conjugation procedure (Lambertsen et al., 2004). To select for gfp-transformed cells, the cellular suspension was plated in R2A medium supplemented with 10% NaCl and the specific antibiotics (Rifampicin 100 μg/ml and Kanamycin 50 μg/ml). The fluorescent-labeling procedure was successful for strains SR7-77 and SR7-87 (both belonging to the *Pseudomonas* genus), as visualized by fluorescence microscopy (Leica). The two *gfp*-labeled strains were selected for colonization experiments on *S. strobilacea* plantlets. A *gfp*-labeled *E. coli* (Favia et al., 2007) was also used as control in order to evaluate the recolonization ability of a non-rhizospheric bacterium. *E. coli* was grown on LB in presence of 100 μg/mL kanamycin. Plants were grown in non-sterile soil microcosms for 6 months starting from seeds. After this period, plantlets were gently harvest, washed, and transfer in pots containing 150 ml of sterile marine water. After 48 h of incubation at 30°C, bacterial cells have been counted and a total of 10^7^ cells/mL have been added to the different pots. The pots were covered with aluminum foils and transferred in the growth chamber (temperature 25°C, 55% humidity). Only the shoots of plants were exposed to the light to permit the normal photosynthetic activity of plants. At successive times (48 and 96 h), the roots bacterized with *gfp*-labeled and the control strains were gently washed to remove the non-attached bacterial cells and analyzed by confocal laser-scanning microscopes (Leica TCSNT) with the Leica Confocal Software. The Leica/EGFP (excitation at 594 nm) and UV (excitation length at 405 nm) have been used to visualize bacterial cells and to reveal the plants tissues shape, respectively. To quantify the colonization ability of the selected strains root and shoot tissues of treated plants were randomly collected, gently washed, and smashed in physiological solution. Serial dilutions have been prepared and plated on petri dishes containing R2A medium (SR7-77, SR7-87, and *no-treated* control) or LB (*E. coli*) supplemented with the required antibiotics and Cycloheximide (100 μg/mL). After 24 h of incubation at 30°C the CFUs per gram were determined.

### Plant Growth Promotion of *S. strobilacea* Plantlets in *In vivo* Assay

*Salicornia strobilacea* scions of 4 cm were obtained and planted in commercial soil. After 1 week of adaptation, the scions were treated with the selected bacterial strains: SR1-55, SR1-57, SR7-77, SR7-82, SR7-83, and SR7-87. Pots were inoculated with a bacterial suspension of 10^7^ cells/g of soil; as control (‘*no treated’*) plantlets were watered with sterile water. Plants were growth in the green house (~ 100 μmol photons m^-2^s of light for 12 h during the day and average temperature of 25°C) and irrigated with sterile marine water once per week. The *in vivo* experiment continued for 110 days after bacterization. After that period the biomass parameters were measured for five replicates per treatment. Fresh and dry weight of shoot and root, length of shoot and root, and number of branches were statistically analyzed using the Student *t*-test, by comparing the data of the different treatments with the ‘*no treated*’ controls.

## Results

### Phylogenetic and Functional Diversity of Cultivable Rhizosphere Bacteria from *S. strobilacea* Subjected to Different Tidal Regimes

**FIGURE 1:**
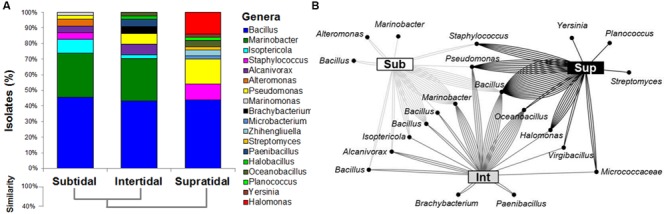
**Phylogenetic affiliation of cultivable bacteria associated to the rhizosphere of *Salicornia strobilacea* grown under different tidal regimes. (A)** Bacterial isolates classification using the RDP classifier at genus level. **(B)** Network analysis dissecting the distribution of the abundance of identified OTUs (97%, dots) associated to the rhizospheric soil (lines) among the three tidal zones (squares).

No significant differences were recorded in the total counts of cultivable bacteria associated to rhizospheric soils subjected to the three different tidal regimes, but a drastic reduction of the CFU/g of soil was observed using the medium added with 20% NaCl (Supplementary Figure 3). The 140 bacterial isolates were de-replicated by strain typing through ITS fingerprinting resulting in 100 different haplotypes. The phylogenetic affiliation of the 16S rRNA gene partial sequences and the network analysis on the dataset are reported in **Figures 1A,B**, respectively. Cluster analysis indicated that the supratidal rhizosphere cultivable bacterial community differed significantly from those collected in the subtidal and intertidal zones (similarity 37.6%), whereas the latter two shared high similarity (65.3%) (**Figure 1A**). The occurrence of specific bacterial assemblages in the *S. strobilacea* rhizosphere subjected to the three different tidal regimes was confirmed by Principal Coordinates Analysis of the ARISA dataset of the whole bacterial community (PERMANOVA, *p* = 0.026, Supplementary Figure 4A), with the subtidal samples showing the higher intra-station variability (Supplementary Figures 4A,B; Supplementary Table 1). Most of the ARISA peaks detected in the environmental DNA extracted from *S. strobilacea* rhizospheres were shared between all stations (91 ARISA peaks, Supplementary Figure 4C).

*Firmicutes* (53%), *Gammaproteobacteria* (39%), and *Actinobacteria* (8%) were the phyla present in the bacterial collection, mainly represented by the genera *Bacillus* (44%), *Pseudomonas* (8%), and *Isoptericola* (4%), respectively. In the three tidal zones a similar abundance was observed only for the genus *Bacillus*, the main bacterial genus isolated from the three rhizosphere samples (**Figure 1A**). However, network analysis revealed that a single OTU within the genus *Bacillus* was commonly present in all samples, whereas other three *Bacillus* OTUs were shared only between subtidal and intertidal samples (**Figure 1B**). The remaining portion of the bacteria collection had a specific composition according to the isolation from the different tidal regimes. For the two areas constantly or cyclically submerged by seawater the second dominant group was represented by the genus *Marinobacter* (28 and 27%, respectively), while in the supratidal area *Pseudomonas*, *Halomonas*, and *Staphylococcus* coexisted in about equal proportion (16, 14, and 10%, respectively). Interestingly, no *Marinobacter* was isolated from the supratidal *S. strobilacea* rhizosphere. The number of *Pseudomonas* isolates remarkably decreased at the increasing distance from the dry area, passing from 16% in supratidal to 7 and 2% in the intertidal and subtidal rhizosphere soils, respectively. The network analysis indicated that the majority of the OTUs present in the supratidal rhizosphere were more abundant in this area respect to the others (**Figure 1B**), pointing out that supratidal rhizosphere host the highest number of specific OTUs respect to the other two zones. The differences observed in the enrichment of different bacterial genera according to the tidal regimes were showed also in Supplementary Figure 5, where the results are presented separately for the two isolation media used in this study (R2A 5% NaCl and KB).

**FIGURE 2:**
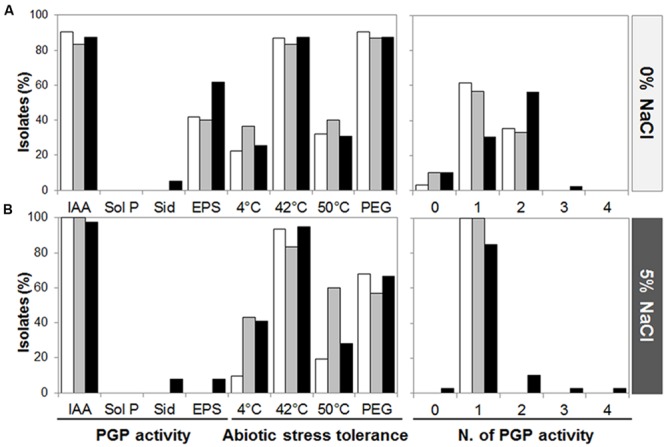
**Plant growth promoting traits and abiotic stresses tolerance of *S. strobilacea* rhizosphere-associated cultivable bacteria.** PGP potential abilities exhibited by each strain have been evaluated in absence **(A)** and presence **(B)** of salt (5% NaCl). IAA, auxin production; Sol P, inorganic phosphate solubilization; Sid., siderophore production; EPS, exopolymers release; PEG, polyethylene glycol. White, gray and black bars indicate subtidal, intertidal, and supratidal samples, respectively.

We assayed one representative of each ITS fingerprinting haplotype for the ability to grow in media supplemented or not with sodium chloride (5 or 10%). All the strains were able to grow on media without or with 5% of NaCl, while only a proportion (56%) of them could grow in presence of 10% NaCl (Supplementary Figure 6A). For this reason, the functional screening was conducted in absence (0%) or presence (5%) of salt (**Figures 2A,B**, respectively). Most of the isolates from *S. strobilacea* rhizosphere were capable of producing IAA-like molecules both in absence and presence of salt (Supplementary Figure 6B). This activity was similarly spread in the three stations (90.3–100% in sub-, 83.3–100% in inter-, and 87.2–97.4% in supra-tidal samples, where the two numbers refer to the presence of 0 or 5% NaCl). EPS production was detected only when bacteria grew in absence of salt and it was present in a lower percentage of the isolates 41.9% in sub-, 40% in inter-, and 61.5% in supra-tidal samples. None of the isolates from the three types of samples presented phosphate solubilization capacities, while siderophore release was detected only in isolates from the supratidal zone in media without (two strains) and with (three strains) NaCl in the growth medium (Supplementary Figure 6B). The abiotic stress tolerance was equally distributed along the tidal gradient and was not driven by the presence of NaCl in the growth medium (**Figures 2A,B**). Even though some activities were quite conserved in the collection, in general the isolates presented a relative low number of PGP activities both in presence and absence of NaCl (99% of isolates had less than two activities) but they showed capacity to tolerate abiotic stresses (**Figures 2A,B**).

### Selection of Isolates and Assessment of the *S. strobilacea* Growth Promotion Ability

**FIGURE 3:**
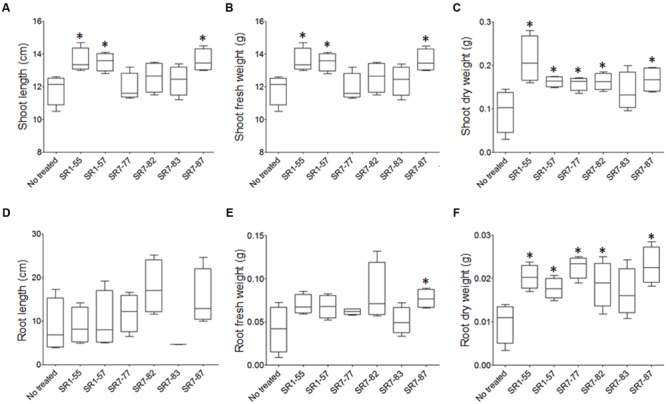
***S. strobilacea* rhizospheric bacteria promote the growth of *S. strobilacea* plantlets in artificial salt system.** Plant biomass after 110 days growth, expressed as mean of shoot **(A)** and root **(D)** length ± standard error; shoot **(B,C)** and root **(E,F)** fresh and dry weights ±standard errors. The data were calculated as average of five plants per treatment and Student *t*-test was adopted to statistically analyze the data. The star (*) indicates statistically significant differences (*p* < 0.05) compared to the ‘no treated’ control.

A cluster analysis performed by combining the bacterial PGP and abiotic stress tolerance traits grouped the strains in four clusters (Supplementary Figure 7). Six strains (SR1-55, SR1-57, SR7-77, SR7-82, SR7-83, and SR7-87) have been selected for the *in vivo* PGP experiments according to the following criteria: (i) belonging to all of the three major clusters, (ii) belonging to the main taxonomic groups (*Bacillus* and *Pseudomonas*), and (iii) isolation from rhizosphere soils of the two extremes of the tidal gradient, the subtidal and supratidal stations. The phylogenetic affiliations and the functional traits of these strains were resumed in the Supplementary Table 2. After 110 days-long experiment, plants exposed to the bacteria and their controls were harvested and analyzed. Plantlets inoculated with strains SR1-55, SR1-57, and SR7-87 showed shoots significantly longer than non-treated ones (**Figure 3A**). The same strains incremented plant fresh and dry shoot biomass (**Figures 3B,C**). While these strains significantly decreased water loss in the plant aerial part (Supplementary Figures 8A,B), strains SR7-77 and SR7-82 incremented the shoot tissue dry weight (**Figure 3C**). Similarly, dry weight increment has been detected in the root system for all the tested strains except SR7-83 (**Figures 3D–F**), confirming that the strains promoted plant growth. No statistical differences from the non-treated plant controls were observed for root length (**Figure 3D**) or the number of branches (Supplementary Figure 8C).

**FIGURE 4:**
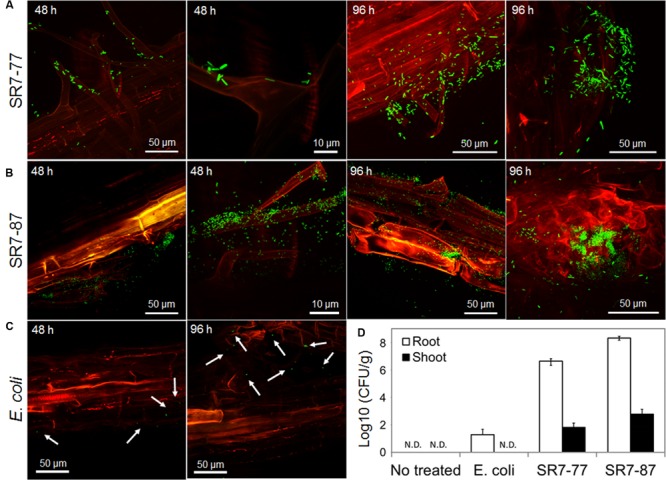
**Rhizocompetence and recolonization ability of *S. strobilacea* rhizospheric strains.** Colonization of *S. strobilacea* root system after 48 and 96 h by three *gfp*-labeled strains: SR7-77 **(A)**, SR7-87 **(B)**, and *E. coli* **(C)**. The *gfp* fluorescence is visible in green. The red color represents the auto-fluorescence of root tissues when excited by the UV laser. Images were obtained with a Leica confocal microscope. **(D)** Re-isolation experiments showing the ability of *Pseudomonas* strains SR7-77 and SR7-87 to actively colonize both root and shoot tissues of *S. strobilacea* plantlets in saline water. Colony forming units (CFU) *per* g of fresh plant tissue are expressed as a mean ± SD of three replicates.

We succeeded in labeling with *gfp* protein two PGP isolates affiliated to the *Pseudomonas* genus, strains SR7-77 and SR7-87. No fluorescent signals were observed in the ‘non-treated’ control (Supplementary Figures 9A,B). The selected *gfp*-labeled strains were able to establish already after 48 h a strong association with the root hairs (**Figure 4A**; Supplementary Figure 10), while *E. coli*, a bacterium not adapted to the rhizosphere, did not effectively recolonize the root and only few cells were observed along the principal and secondary root or on root hairs (**Figure 4C**; Supplementary Figures 9C,D). Both the *Pseudomonas* strains colonized the root starting from root hairs or following the surface of the connection points between principal and secondary roots, where the active growth of the tissues create favorable niche of colonization (**Figures 4A,B**; Supplementary Figure 10). The microscopy analysis conducted after 96 h confirmed the ability of both strains to stably colonize the root (**Figures 4A,B**). Re-isolation of the inoculated *gfp*-tagged strains from plant tissue confirmed the microscope observations. Both *Pseudomonas* strains were recovered at high titres from the plantlets especially from the root, indicating that they actively grow in the rhizosphere. Densities ranging from 4.25 × 10^6^ to 2.11 × 10^8^ CFU per gram of tissue were observed in the root tissues, while few colonies were counted in the *E. coli* plates (1.95 × 10^1^) and no colonies were present in the negative control ones (**Figure 4D**). A lower number of CFU per g was observed in the shoot tissues with both the bacteria (6.49 × 10^1^ in SR7-77 and 6.09 × 10^2^ in SR7-87, **Figure 4D**) indicating that they are capable to move from the root to the shoot. From all treatments, no rifampicin/kanamycin-resistant isolates other than strains SR7-77, SR7-87, or *E. coli* were obtained from the inoculated plants, while no resistant colonies were obtained from non-inoculated control plants, indicating that no contamination by spontaneous rifampicin/kanamycin-resistant bacteria occurred.

## Discussion

### Tidal Regime Shapes Bacterial Communities’ Structure Maintaining the PGP Potential of *S. strobilacea*-Associated Bacteria Unvaried

In coastal habitats subjected to significant tidal ranges, the marine water flow enhances the turnover of nutrients and organisms, including the sediment microbiome. For instance, in mangrove ecosystems, exposure to tidal flow influences the microbial growth and biomass in intertidal surface sediments (Alongi, 1988). Under the tidal flow bacteria naturally associated to plant roots are subjected to periodical changes of selective pressures, on top of those constantly imposed by the sediment and root exudates. Even though such a continuous environmental pressure variations is foreseen to influence the structure and functionality of the rhizosphere communities (Haichar et al., 2008; Lareen et al., 2016), investigation on the shifts of PGP microbial communities across a gradient of tidal inundation has been overlooked. Our study assessed the effects of tide on plant-associated microbiome diversity and functionality, by considering the structure of the PGP bacterial community associated to the rhizosphere of *S. strobilacea* plants growing in subtidal, intertidal, and supratidal sediments. We moved beyond the consideration of the sole intertidal zone: we selected a constant environment represented by the rhizosphere of *S. strobilacea*, a plant species that grows under all tidal conditions detected along the tide gradient in order to evaluate the tide-effect on PGP bacterial functionality.

Although subjected to the rhizosphere effect and thus showing a core pool of microbial isolates, *S. strobilacea* hosted rhizospheric bacteria whose distribution was strongly determined by tidal regimes occurring in the three zones. Such uneven distribution of bacterial isolates could be explained by the different oxygen availability in the subtidal zone, strictly related to the growth pattern of root, from which oxygen can be lost providing a more suitable surrounding environment for microbes (Berg and Smalla, 2009; Oliveira et al., 2014). In coastal lagoon and salt marshes, halophytes like *Spartina maritima*, *Sarcocornia perennis*, and *Halimione portulacoides*, differentially influence the activity and distribution of microbial populations through their diverse growth and resources allocation (Oliveira et al., 2010, 2014). Similar results were obtained by Borruso et al. (2014) investigating the rhizospheric bacterial community of *Phragmites australis* in different sites located in a hypersaline pond. Furthermore, a correlation between the bacterial communities associated to *S. perennis* and *H. portulacoides* specimens and the site of plant collection was reported in salt marsh sediments (Oliveira et al., 2014). We are aware of the limitations of the cultivation-based approach (for instance, we focused on the fast-growing bacteria that grow forming colonies after 72 h of incubation and we have not considered the slow growing bacteria). However, we found that the distribution of the phylogenetic affiliation of bacteria isolated from *S. strobilacea* rhizosphere was in agreement with the diversity described by the ARISA molecular dataset. No differences in term of total CFU per gram of rhizospheric sediment were observed in the three areas. At the phylogenetic level, *Firmicutes*, *Actinobacteria*, and *Gammaproteobacteria* were highly abundant phyla in the *S. strobilacea* rhizosphere in all the three tidal zones, in agreement to what already reported in other saline ecosystems of both marine and terrestrial origin (Tang et al., 2011; Mapelli et al., 2013a; Marasco et al., 2013a; Oliveira et al., 2014; Bargiela et al., 2015a,b; Soussi et al., 2016; and reference therein). The genus *Bacillus*, well known for its interaction with plants (Raddadi et al., 2007; Marasco et al., 2012), was the prevalent genus in all tidal zones, but many other genera were selectively enriched by the different tidal conditions. *Marinobacter*, for example, was highly abundant in the subtidal and intertidal rhizospheres flooded by seawater. *Marinobacter* is a halophilic genus typically found in seawater and marine sediments (McGenity et al., 2012; Amer et al., 2015), as is the *Alcanivorax* genus (Head et al., 2006; McGenity et al., 2012), which representatives were also isolated exclusively from subtidal and intertidal rhizospheres. In the supratidal rhizosphere, *Marinobacter* and *Alcanivorax* were replaced by salt-loving bacteria of the genus *Halomonas*, which have been previously observed in association with *Salicornia* spp. (Argandoña et al., 2005; Jha et al., 2012; Mapelli et al., 2013a) and other halophytes (Borruso et al., 2014). Together with *Bacillus*, the genus *Pseudomonas* was one of the two genera isolated using both the media and overall its abundance was higher in the supratidal rhizospheres. Both genera are colonizing the rhizosphere of several cultivated and wild plants under stressful conditions such as salinity and drought (Marasco et al., 2012; El-Sayed et al., 2014; Rolli et al., 2015).

All the data obtained demonstrated that plants growing across the tide regimes selected specific community at OTUs level. The network analysis disentangled intra-genus variability with specific OTUs associated to different tidal regimes occurring in the studied gradient. This is the case also for *Bacillus*, the prevalent genus in all sites, which comprised both OTUs shared by *S. strobilacea* growing under all tidal conditions and other typical of subtidal samples.

Despite the different bacterial phylogenetic composition in the three tidal areas, the investigated phenotypic traits related to PGP potential did not significantly change according to our initial hypothesis. This result is similar to our previous observation on the bacterial PGP potential of grapevine growing under different climate and soil conditions (Marasco et al., 2013b) while it was recently shown that specific agronomic practice, like biochar addition to the soil, influences the bacterial community on both the taxonomic diversity and the expression of PGP traits (Egamberdieva et al., 2016). Compared to previous studies, bacteria associated to *S. strobilacea* rhizosphere did not present many PGP activities under the tested conditions (Mapelli et al., 2013a; Mesa et al., 2015). However the ability to tolerate the abiotic stresses, such as salinity, osmotic stress, and temperature variation, was widespread in the collection, confirming the adaptability of these strains to stressors typical of the environment of origin. Among the toxic effects caused by high salinity, the reduction of nutrients availability (i.e., P, Fe, N), caused by precipitation and immobilization, represents an additional problem for plant and microorganism survival (Shrivastava and Kumar, 2015). Several works demonstrated that bacteria are able to solubilize nutrients making them available for the bio-uptake (Mapelli et al., 2012). Beyond a biofertilizing activity, bacteria can be also involved in the activation of the plant Induced Systemic Tolerance (IST), phytohormones production that favor plant development (Yang et al., 2009). In our study, only few isolates showed potential for bio-fertilization activity, such as production of siderophore or solubilization of phosphate, (Vessey, 2003; Mapelli et al., 2012; Shrivastava and Kumar, 2015). On the contrary, almost all isolates were able to produce IAA *per se*, reaching the 100% of producers when salt was added to the medium. The induction of IAA production under salt condition was recently documented by Yaish et al. (2015) emphasizing the role of phytohormones produced by root associated PGP bacteria, especially under adverse conditions. IAA is a plant hormone, belonging to the auxin group, which plays many different roles in plant growth and development. While at cellular level auxins are involved in the regulation of cell division and elongation, at whole plant levels they contribute mainly to roots development (Simon and Petrášek, 2011), also in response to salinity (Iglesias et al., 2010, 2011). Even though auxins are phytohormones produced by plants, IAA-producing bacteria are able to interact with the plant auxin pathways in their own favor establishing a host-beneficial microorganism’s interaction that often, depending on the plant and the conditions, determine an increased plant growth (Dimkpa et al., 2009). Auxin production is widespread among halotolerant PGP bacteria belonging to several genera, such as *Bacillus*, *Brevibacterium*, *Achromobacter*, *Brachybacterium*, *Pseudomonas*, and *Halomonas* (Egamberdieva, 2009; Sgroy et al., 2009; Piccoli et al., 2011; Jha et al., 2012; Mapelli et al., 2013a; Yaish et al., 2015). During salt stress, IAA-producing bacteria are involved in alleviating salinity-induced dormancy showing high stimulatory effect on the root and shoot length (Egamberdieva, 2009; Jha et al., 2012; Yaish et al., 2015).

### *S. strobilacea* Rhizobacteria Have the Potential to Harness Their PGP Benefits Effect *In vivo*

Despite the bacteria isolated did not present several PGP traits *in vitro*, they were able to support plant growth confirming that bacterial strains were able to enhance plant growth under saline conditions, presumably through IAA production as previously shown in absence of salt (Patten and Glick, 2002). The selected bacteria mainly showed effects on the plant promotion at the shoot level. In particular, strains SR1-55, SR1-57, and SR7-87 increased both length and biomass of aerial parts favoring accumulation of water in the tissues. Differently, strains SR7-77 and SR7-82 caused an increase of plant dry weight rather than water content, showing the capacity of stimulating tissues biomass.

The main biostimulators identified in this study belong to the *Pseudomonas* and *Bacillus* genera. These genera have been already characterized in arid and saline ecosystems for their capacity to resist to multiple abiotic stresses, to grow in association with plant both with a rhizospheric and endophytic life-style and to exert PGP activities involved in the promotion and protection of plant growth (Marasco et al., 2012; Cherif et al., 2015). The strains able to promote the growth were deriving from the rhizosphere of plants both from intertidal and supratidal zones indicating that *S. strobilacea* is capable of recruiting PGP bacteria independently from the tidal conditions.

Before bacteria can express any PGP service, they must stably colonize the root system and finally spread throughout the plant’s tissues (Rodriguez et al., 2008; Brader et al., 2014; Hardoim et al., 2015). *Pseudomonas* stains *gfp*-SR7-77 and *gfp*-SR7-87 efficiently colonized *S. strobilacea* plants, starting from the root system and moving to the aerial parts. The capacity of moving between different organs of the plant (for instance from the root to the shoot) should be further investigated to assess if the bacteria move through the endosphere, the surface of the plant tissues or through aerosols from the rhizosphere. The two selected strains belonged to the same genus but presented a different PGP potential both *in vitro* and *in vivo*, confirming the importance for the plant to interact with specific bacteria (Panke-buisse et al., 2015). The selected strains provided to the host-plant ecological services useful for plant adaptation, growth, and development, particular in saline soil (Daffonchio et al., 2015).

## Conclusion

The role of tidal regime on shaping the structure of rhizosphere bacterial communities has been demonstrated. Despite the phylogenetic difference of bacterial community composition, *S. strobilacea* growing across different tidal regimes enriched bacteria able to (i) resist to an array of abiotic stresses typical of extreme environments, (ii) produce plant hormones, and (iii) stably colonize plant root. Overall, these characteristics confer to the isolated strains all necessary adaptive traits to explicate their PGP activity in saline soils. In this context, the ability to enrich and recruit PGP bacteria associated with salt-adapted plant is a promising research area to be further developed in upcoming years, and a key aspect for the selection novel PGP candidates (Philippot et al., 2013).

## Author Contributions

Conceived and designed the experiments: RM, FM, ER, SB, and DD. Performed the experiments: RM, FM, PB, and MM. Analyzed the data: RM, FM, MF, ER, and PB. Contributed to reagents/materials/analysis tools: DD, SB, and MR. Wrote the paper: RM, FM, SB, and DD. Collected the samples: RM, FM, ER, DD, SB, AC, and GT. Critically revised the manuscript: MR, AC, and GT.

## Conflict of Interest Statement

The authors declare that the research was conducted in the absence of any commercial or financial relationships that could be construed as a potential conflict of interest.

## Footnotes

1 https://npgsweb.ars-grin.gov/gringlobal/taxonomydetail.aspx?id=32669

2 http://bioinformatics.psb.ugent.be/webtools/Venn/
