# Supplementary material for: Salicornia strobilacea (Synonym of Halocnemum strobilaceum) Grown under Different Tidal Regimes Selects Rhizosphere Bacteria Capable of Promoting Plant Growth
Source: Front Microbiol. 2016 Aug 22;7:1286. doi: 10.3389/fmicb.2016.01286 (PMC4992691; doi:10.3389/fmicb.2016.01286)
Supplement: Supplementary file 1 [file Data_Sheet_1.DOCX]

**SUPPLEMENTARY MATERIAL TO:**

***Salicornia strobilacea* (Synonym of *Halocnemum strobilaceum*) Grown under Different Tidal Regimes Selects Rhizosphere Bacteria Capable of Promoting Plant Growth**

Ramona Marasco^1#^, Francesca Mapelli^2#^, Eleonora Rolli^2^, Maria J. Mosqueira^1^, Marco Fusi^1^, Paola Bariselli^2^, Muppala Reddy^1^, Ameur Cherif^3^, George Tsiamis^4^, Sara Borin^2*^, Daniele Daffonchio^1,2*^

^1^ Biological and Environmental Sciences and Engineering Division, King Abdullah University of Science and Technology, Thuwal, Saudi Arabia

^2^ Department of Food, Environmental and Nutritional Sciences, University of Milan, Milan, Italy

^3^ Greenhouse Laboratory, King Abdullah University of Science and Technology, Thuwal, Saudi Arabia

^4^ Institut Supérieur de Biotechnologie Sidi Thabet, BVBGR-LR11ES31, University of Manouba, Ariana, Tunisia

^5^ Department of Environmental and Natural Resources Management, University of Patras, Panepistimioupoli Patron, Greece

^#^ Equal contribution

*Correspondence:

Daniele Daffonchio

[daniele.daffonchio@kaust.edu.sa](mailto:daniele.daffonchio@kaust.edu.sa)

Sara Borin

sara.borin@unimi.it

**Running title:** Bacterial PGP services in *Salicornia* under different tidal regimes

**SUPPLEMENTARY TABLES**

**Supplementary Table 1.** Diversity of bacterial community associated to *Salicornia* rhizosphere, as described through ARISA fingerprinting.

| **Index** |  | ***Salicornia* rhizospheric soil** | | | | | | | | |
| --- | --- | --- | --- | --- | --- | --- | --- | --- | --- | --- |
|  |  | **Sub1** | **Sub2** | **Sub3** | **Inter1** | **Inter2** | **Inter3** | **Supra1** | **Supra2** | **Supra3** |
| OTUs |  | 48 | 104 | 108 | 93 | 100 | 124 | 106 | 103 | 111 |
| *Shannon* |  | 3.31 | 4.22 | 4.12 | 3.99 | 4.03 | 3.85 | 4.13 | 3.97 | 4.17 |
| *Dominance* |  | 0.07 | 0.02 | 0.03 | 0.03 | 0.03 | 0.06 | 0.02 | 0.04 | 0.03 |
| *Simpson* |  | 0.93 | 0.98 | 0.97 | 0.97 | 0.97 | 0.94 | 0.98 | 0.96 | 0.97 |
| *Evenness* |  | 0.57 | 0.66 | 0.57 | 0.58 | 0.56 | 0.38 | 0.59 | 0.51 | 0.58 |

Sub = Subtidal; Inter = Intertidal; Supra = Supratidal

1 = rhizosphere of plant 1; 2 = rhizosphere of plant 2; 3 = rhizosphere of plant 3

**Supplementary Table 2.** Bacterial strains selected for the PGP *in vivo* experiments.

| **Tidal Regime** | **Strain** | **Identification^#^**  **(% identity)** | **PGP activity (-/+ NaCl)** | | | |  | **Abiotic stress tolerance (-/+ NaCl)** | | | |
| --- | --- | --- | --- | --- | --- | --- | --- | --- | --- | --- | --- |
|  |  |  | **IAA** | **Sol P** | **Sid** | **EPS** |  | **4°C** | **42°C** | **50°C** | **PEG** |
| **Subidal** | SR1-55 | *B. aquimaris* (99) | 0/1 | 0/0 | 0/0 | 1/0 |  | 0/0 | 1/0 | 0/0 | 1/1 |
|  | SR1-57 | *B. vallismortis* (99) | 1/1 | 0/0 | 0/0 | 0/0 |  | 0/0 | 1/1 | 1/1 | 1/1 |
| **Supratidal** | SR7-77* | *P. stutzeri* (99) | 1/1 | 0/0 | 0/0 | 1/1 |  | 1/1 | 0/1 | 0/0 | 1/0 |
|  | SR7-82 | *B. stratosphericus* (100) | 1/1 | 0/0 | 1/0 | 1/0 |  | 0/0 | 1/1 | 1/0 | 1/1 |
|  | SR7-83 | *M. takaoensis* (98) | 1/1 | 0/0 | 0/0 | 1/0 |  | 0/0 | 1/1 | 0/0 | 1/0 |
|  | SR7-87* | *P. stutzeri* (99) | 1/1 | 0/0 | 0/0 | 1/0 |  | 1/0 | 1/1 | 0/0 | 1/1 |

# Identification was determined according to the partial sequence of the 16S rRNA gene

IAA: auxin production; Sol P: phosphate solubilization; Sid: siderophore production; ESP: exopolysaccharide production.

-/+NaCl: absence/presence of 5%NaCl in the growth media

* *Gfp-*labelled strains used in the salicornia recolonization assay

**SUPPLEMENTARY FIGURES**

**Supplementary Figure 1.** Sampling site was located in the coastal area south of Zarzis **(A)** and magnified image of the selected area **(B)** by Google Earth software. The studied costal area **(C)** was characterized by cyclical sea tide that define three different tidal area where *Saliconia* sp. plants growth: subtidal area, always flooded **(D)**, intertidal area, subjected to cyclical submerging **(E)** and supratidal area, not submerged by the sea water **(F)**. *Salicornia* plants (n. 9) were harvested and the root system sampled **(G)**.

**
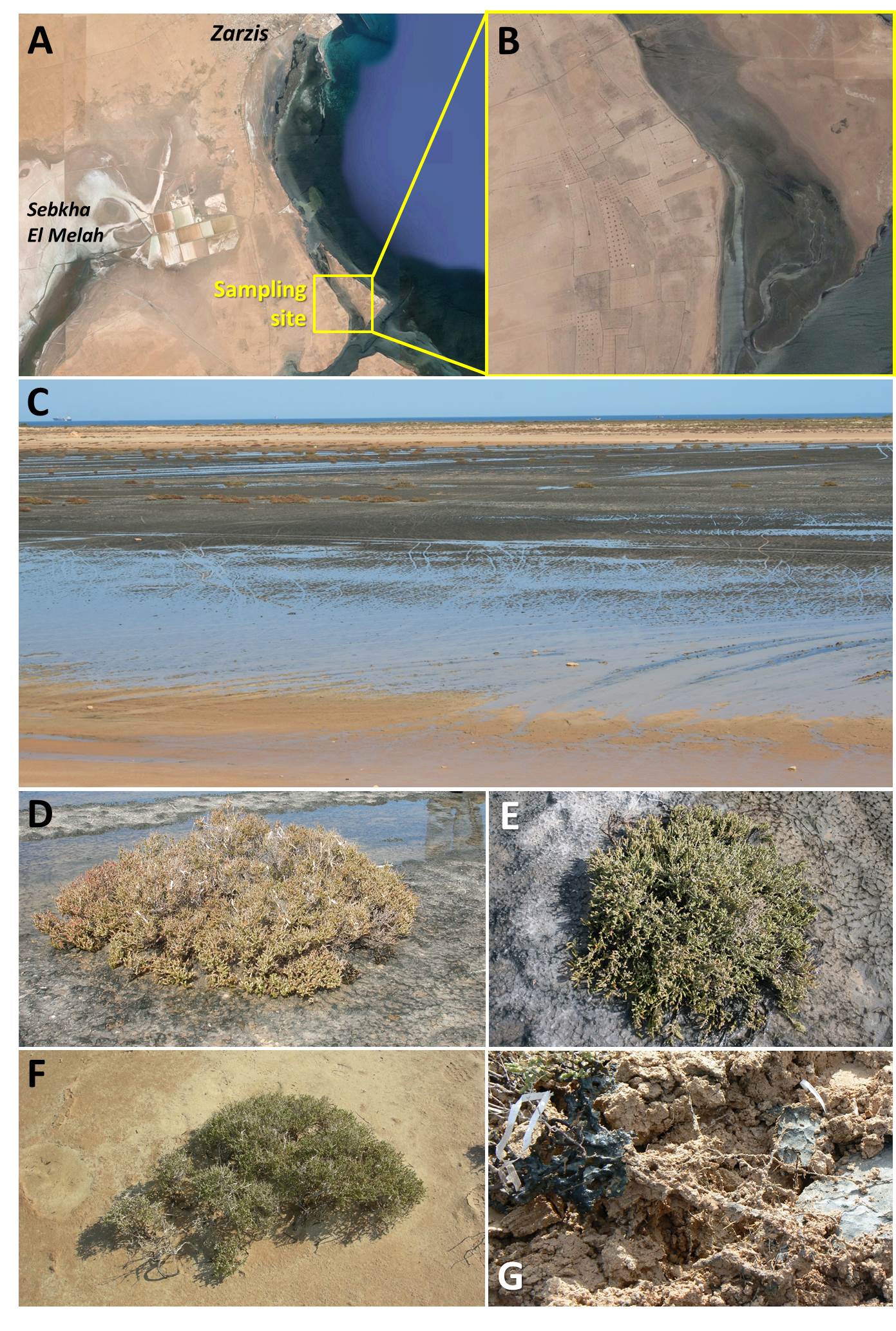
**

**Supplementary Figure 2.** Environmental parameters affecting the sampling site. Temperature, humidity, rainfall and tide variations in 2008.

**
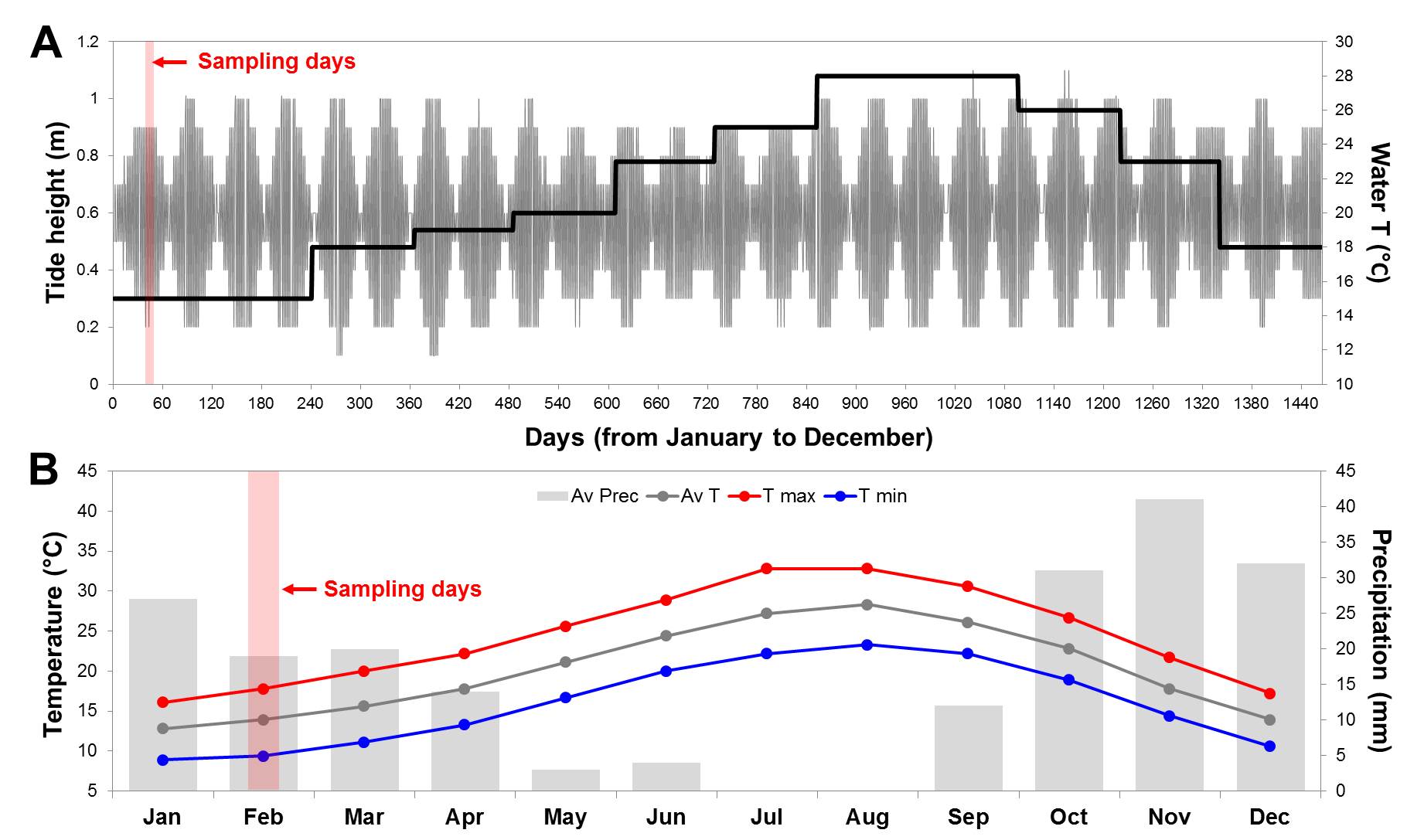
**

**Supplementary Figure 3.** Colony Forming Unit (CFU) calculated per gram of *Salicornia* rhizosphere.

**
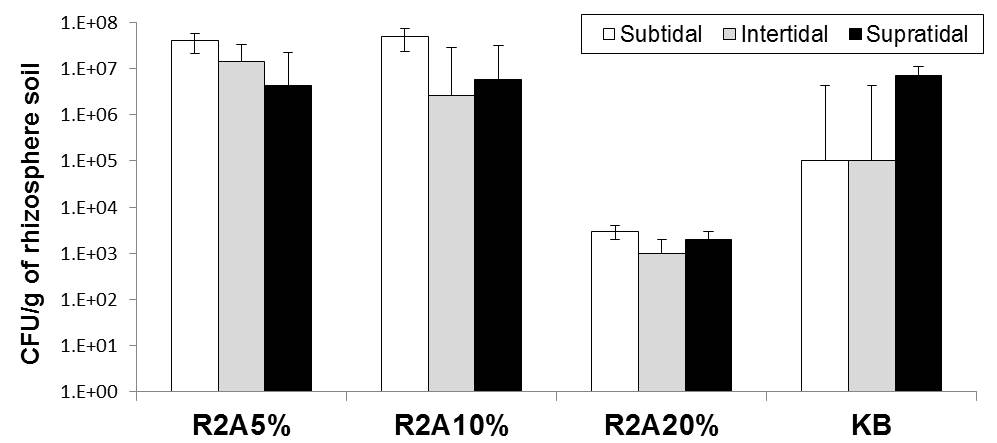
**

**Supplementary Figure 4.** Structure of bacterial community associated to the rhizosphere soil of *Salicornia* plants growing under different tidal regimes. **(A)** Principal Coordinate analysis (PCO) based on ARISA fingerprints. **(B)** Boxplots of diversity (Shannon index, H) calculated from ARISA fingerprints. **(C)** Venn diagram of ARISA OTUs.

**
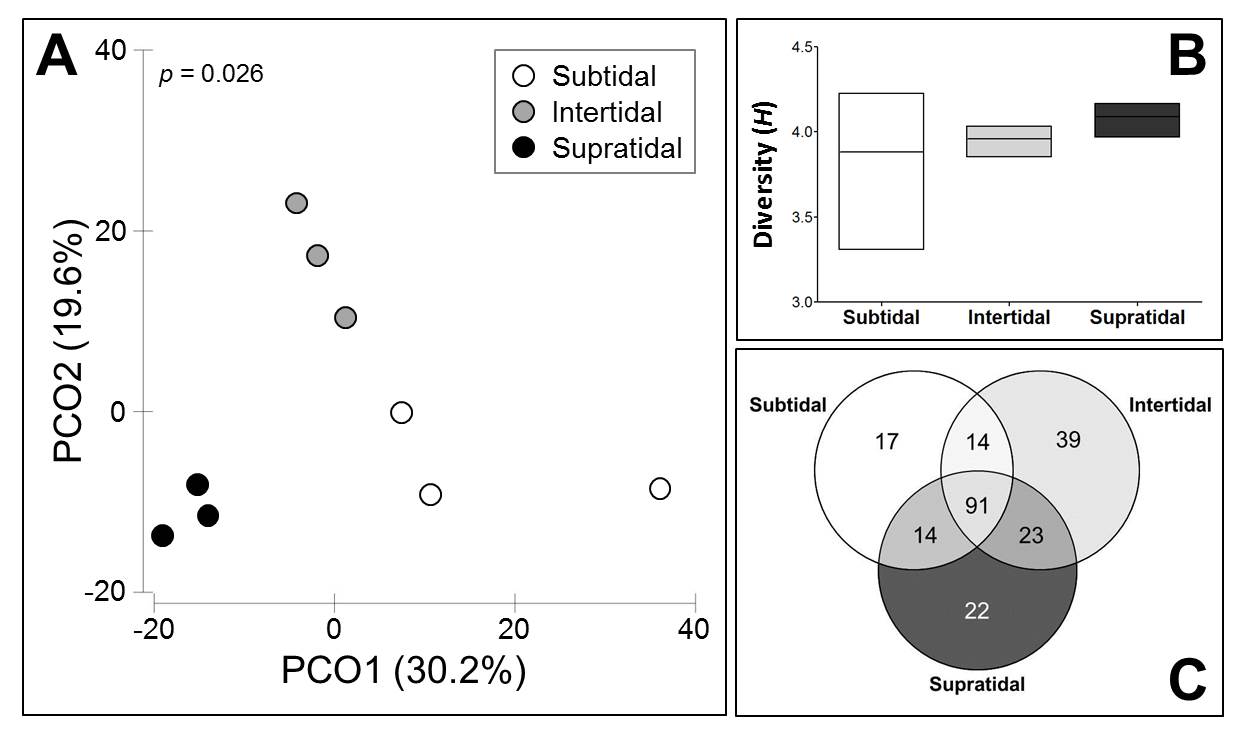
**

**Supplementary Figure 5.** Bacterial isolates classification using the RDP classifier at genus level. Identification of bacteria isolated on the R2A medium added with 5% NaCl **(A)** and on King’s B medium **(B)**


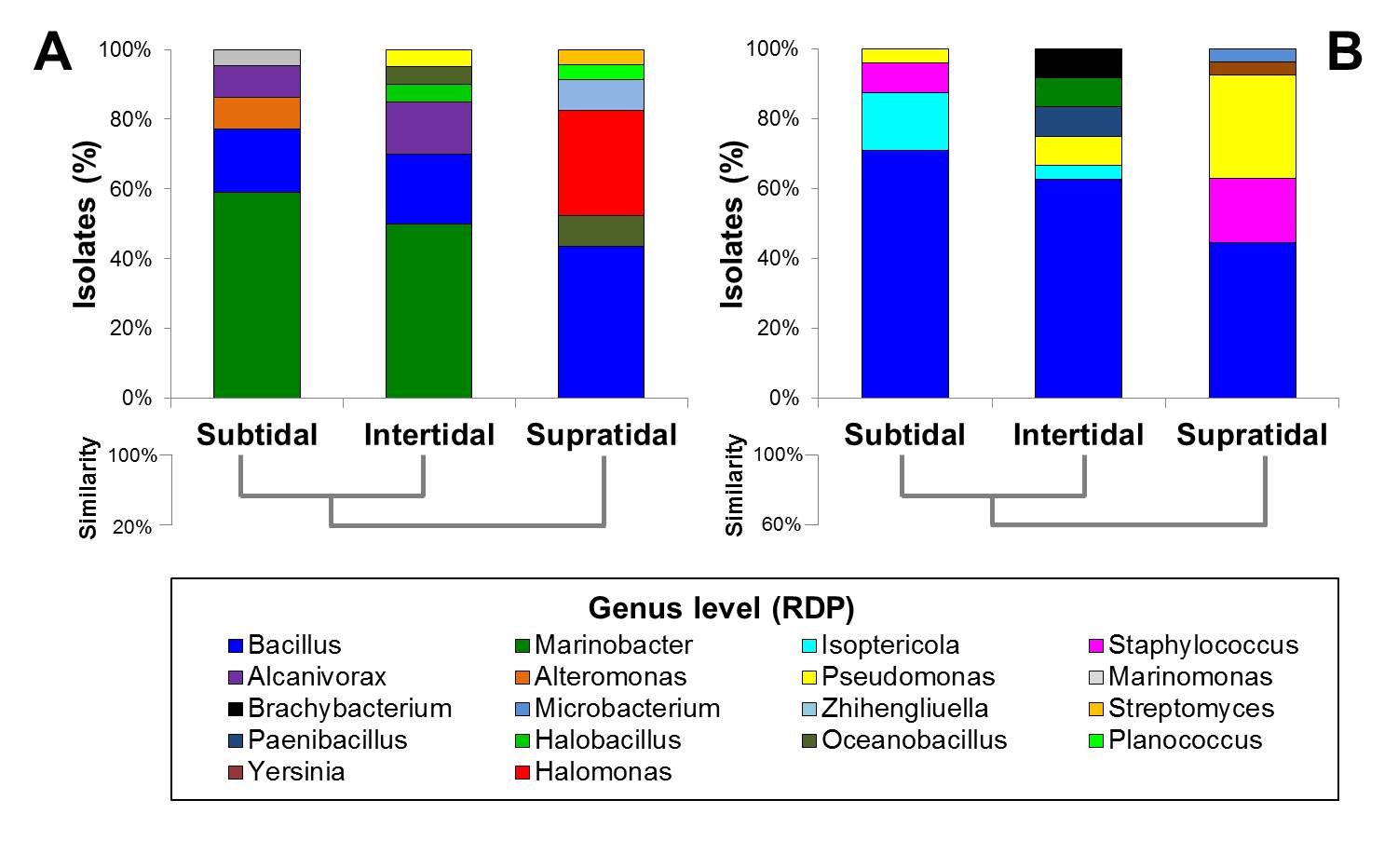


**Supplementary Figure 6.** Heat map of Plant Growth Promoting (PGP) activity and abiotic stress tolerance of the isolated bacterial strains. (A) Salt tolerance adding different concentrations of salt (NaCl) in the growth medium. (B) Evaluation of PGP activity and abiotic stress tolerance in absence (left) and presence of 5% NaCl (right). The tests have been done in triplicate. Black (■) and white (□) squares indicate the presence or absence of the activities.

**
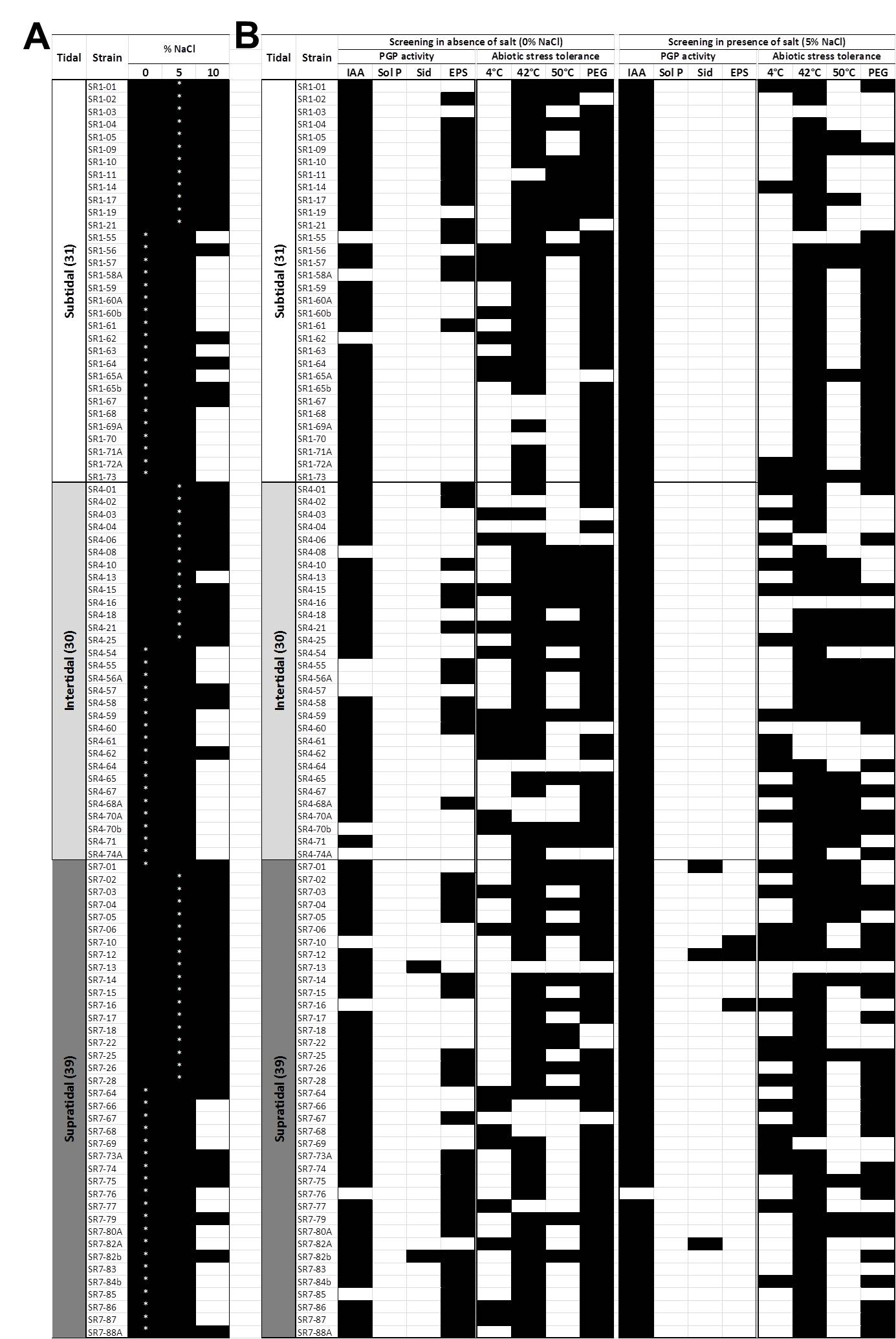
**

**Supplementary Figure 7.** Selection of PGP bacteria for the *in vivo* assay.

**
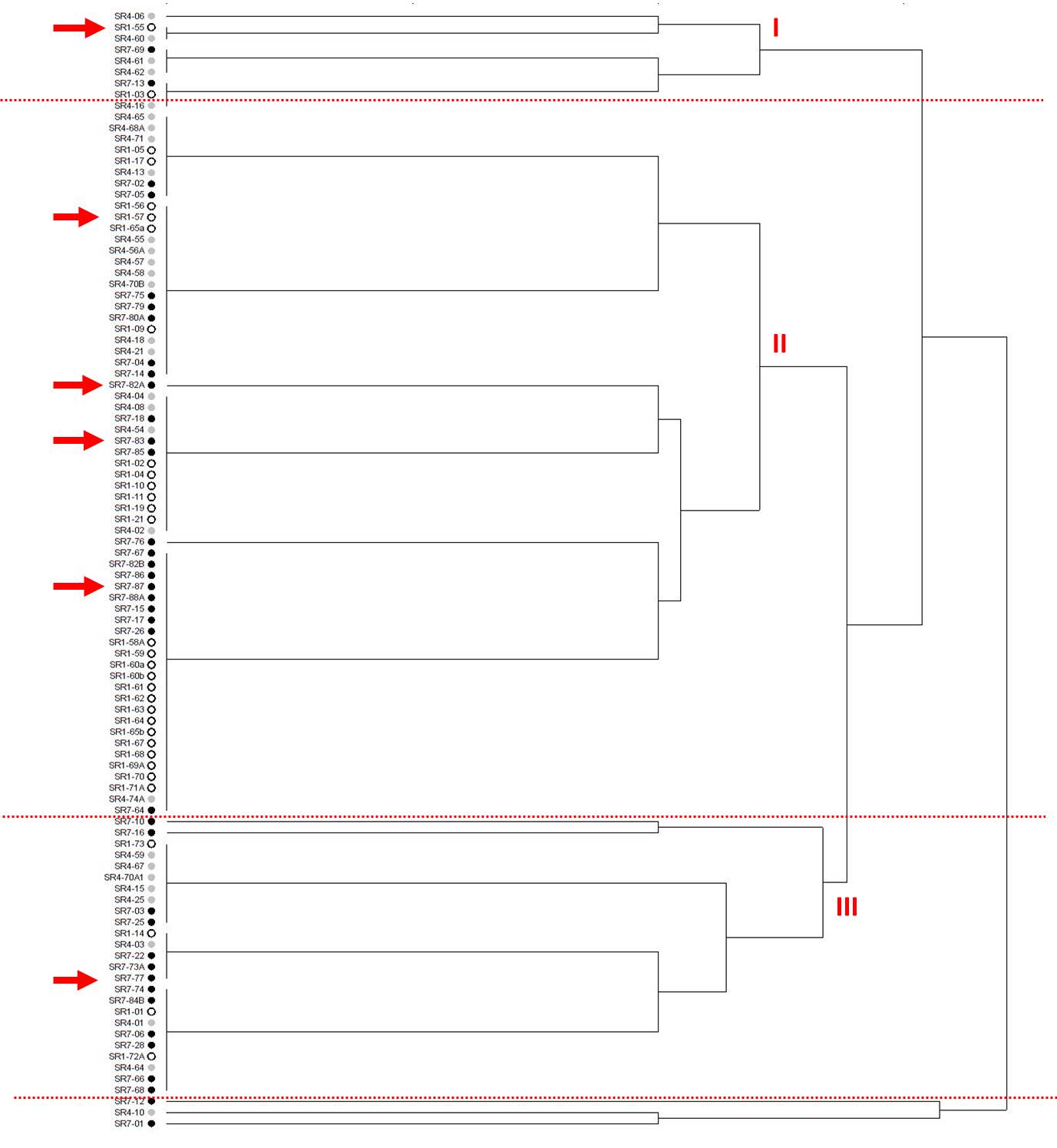
**

**Supplementary Figure 8.** Water content of shoot **(A)** and root **(B)** and number of shoot branches **(C)** of *Salicornia* plants treated with the selected PGP bacteria. The statistical analysis was conducted comparing the data with the non-treated control. All data presented are averages of five replicates. Significant differences were represented by a star (*) with *p*<0.05.

**
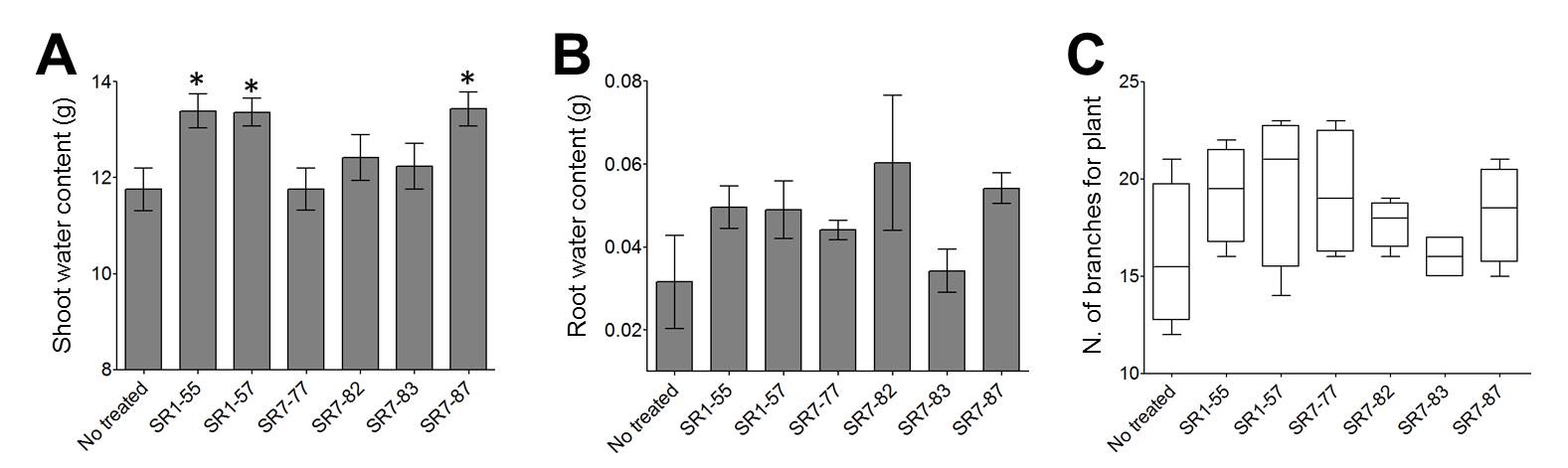
**

**Supplementary Figure 9.** Recolonization assay of *Salicornia* plantlets. Roots were collected from non-treated control plants **(A** and **B)** and *E. coli*-treated plants **(C** and **D)** after 48 and 96 hours. The first column represent the overlay of two different confocal microscope images, i) the red channel indicating the autofluorescence of root tissues excited by UV laser (second column) and ii) the green channel (third column) showing the emission of *gfp*-labelled cells. The fourth column shows the images under bright-field. Arrows indicate the fluorescent *E. coli* cells.


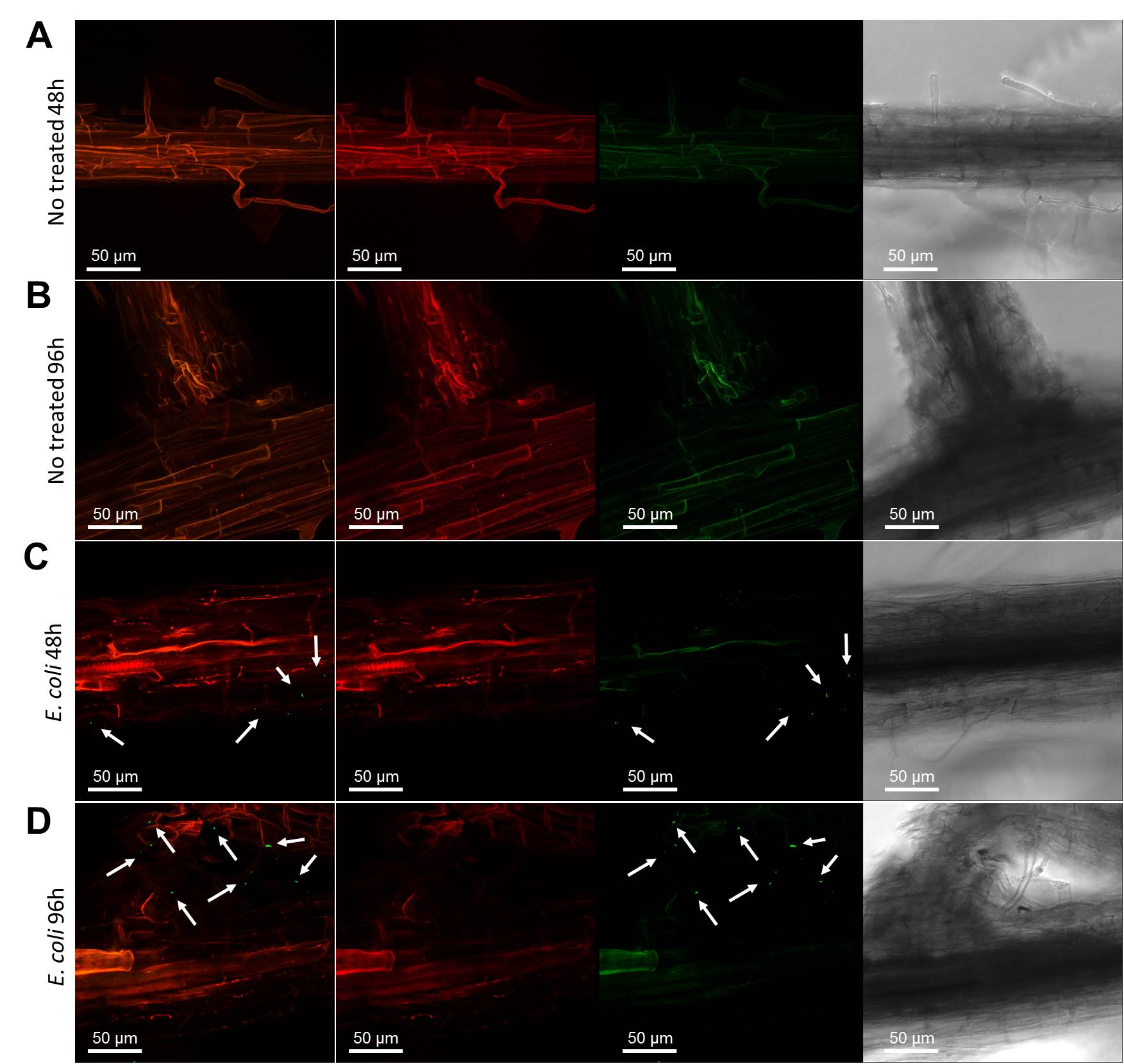


**Supplementary Figure 10.** Recolonization assay of *Salicornia* plantlets with strains SR7-77 **(A)** and SR7-87 **(B)** after 48 and 96 hours. The first column in the figure reports the images of Figure 4 and is accompanied by the following three images showing the two different fluorescence channels and the bright field. The first column represents the overlay of two different confocal microscope images, i) the red channel indicating the autofluorescence of root tissues excited by UV laser (second column) and ii) the green channel (third column) showing the emission of *gfp*-labelled cells. The fourth column shows the images under bright-field.

**
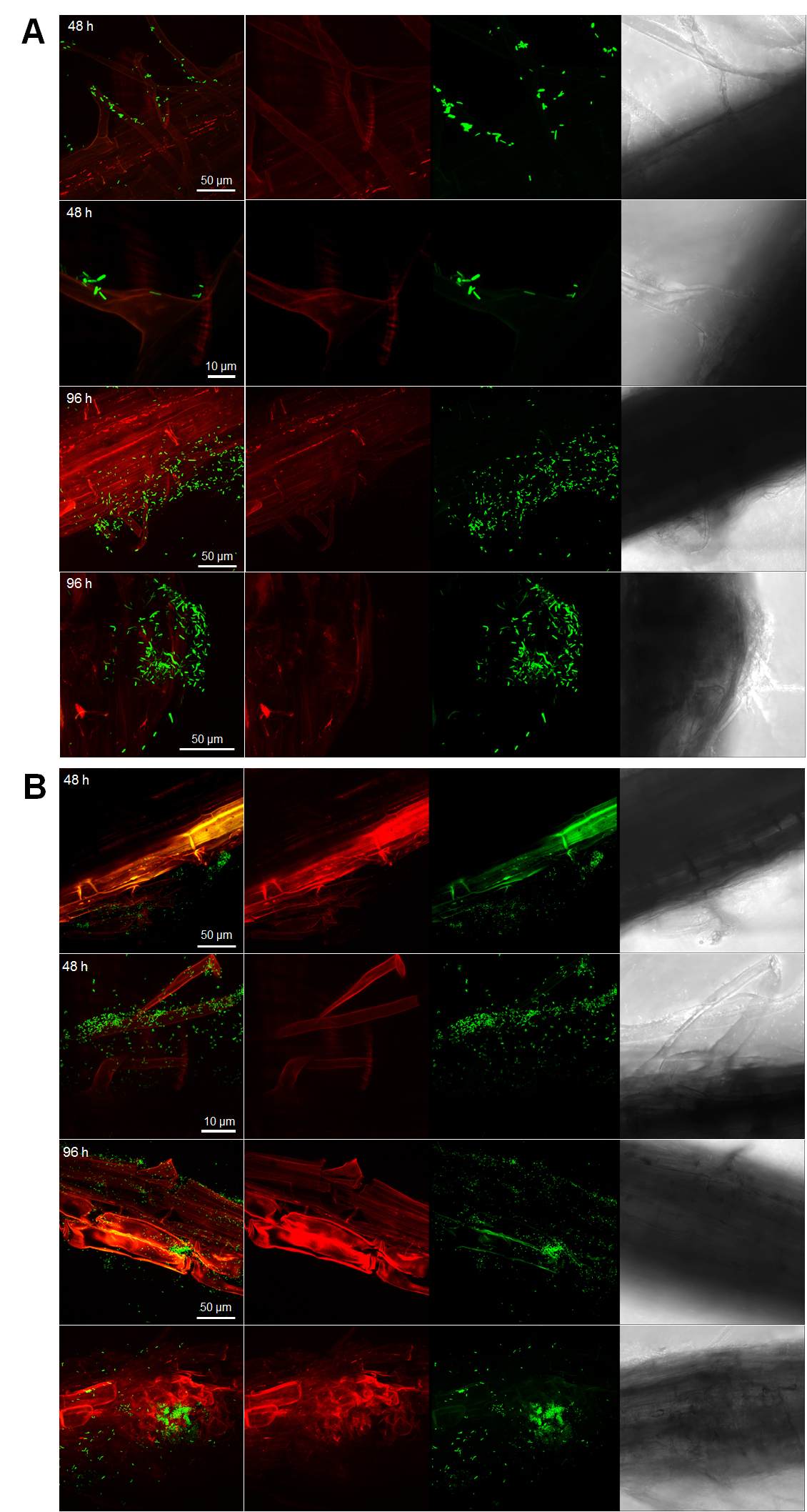
**

**
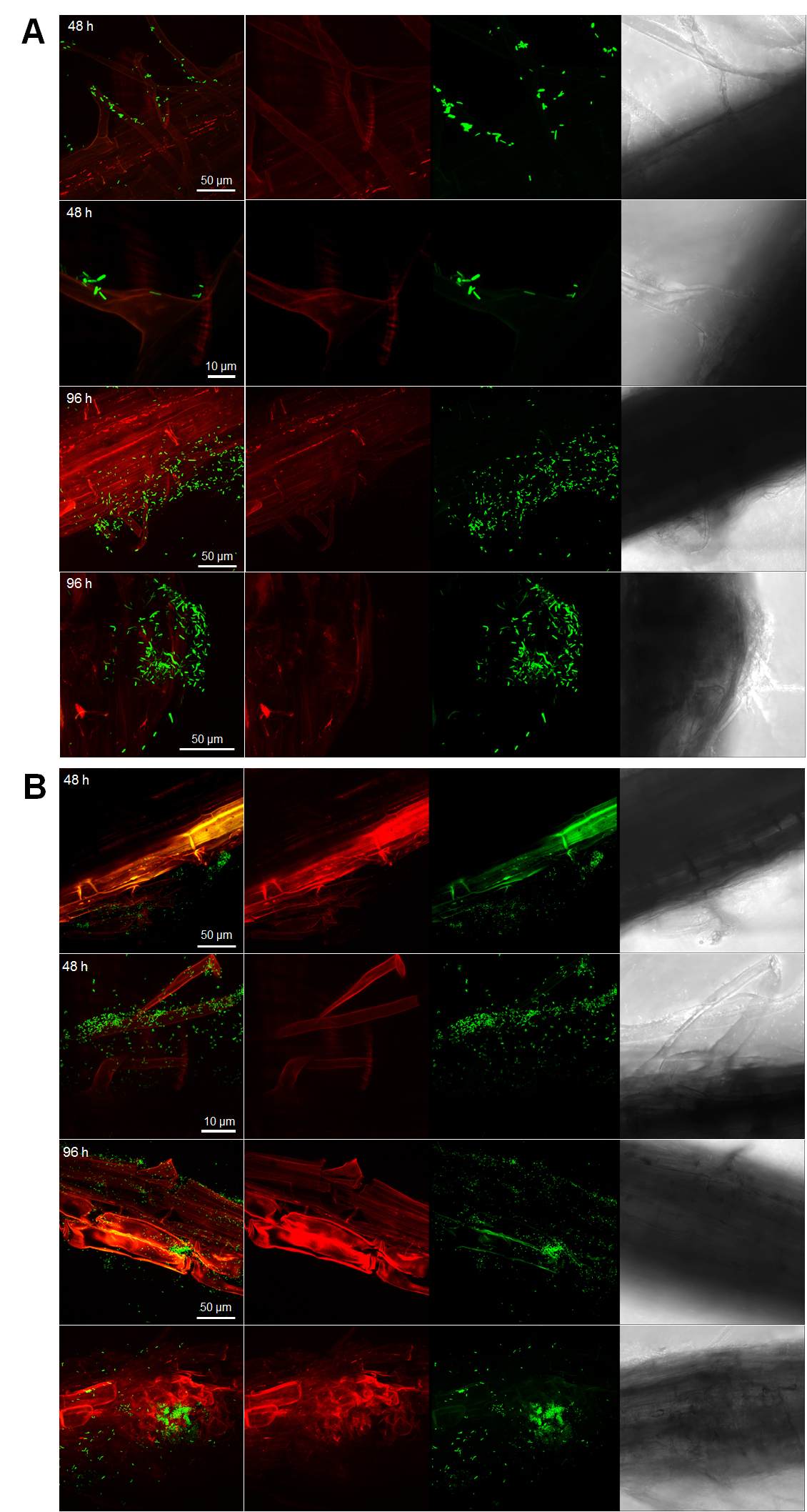
**
